# Judging the Difference between Attractiveness and Health: Does Exposure to Model Images Influence the Judgments Made by Men and Women?

**DOI:** 10.1371/journal.pone.0086302

**Published:** 2014-01-20

**Authors:** Ian D. Stephen, A. Treshi-Marie Perera

**Affiliations:** University of Nottingham Malaysia Campus, Semenyih, Selangor, Malaysia; University of Udine, Italy

## Abstract

Recent research has shown facial adiposity (apparent weight in the face) to be a significant predictor of both attractiveness and health, thus making it an important determinant of mate selection. Studies looking at the relationship between attractiveness and health have shown that individuals differentiate between the two by preferring a lower weight for attractiveness than for health in female faces. However, these studies have either been correlational studies, or have investigated weight perceived from only the face. These differences have been discussed with regard to sociocultural factors such as pressure from parents, peers and also media, which has been seen to have the highest influence. While exposure to media images has been shown to influence women’s own-body image, no study has yet directly tested the influence of these factors on people’s preferred weight in other women’s bodies. Here we examine how a short exposure to images of models influences men’s and women’s judgments of the most healthy looking and attractive BMI in Malaysian Chinese women’s bodies by comparing differences in preferences (for attractiveness and health) between groups exposed to images of models of varying attractiveness and body weight. Results indicated that participants preferred a lower weight for attractiveness than for health. Further, women’s but not men’s preferred BMI for attractiveness, but not health, was influenced by the type of media images to which they were exposed, suggesting that short term exposure to model images affect women’s perceptions of attractiveness but not health.

## Introduction

Selecting a desirable mate of good quality is an important decision made by individuals, as it determines the quality and number of offspring they can produce. It is therefore important that we are sensitive to cues that honestly signal one individual to be more desirable than another [1]. Evolutionary psychological explanations suggest that physical attractiveness cues health and fertility, and therefore enhances mating success in humans [2]. A preference for such attractive traits is therefore important in determining a mate of good quality [3]–[4].

Body mass index (BMI, weight scaled for height) and, to a lesser extent, waist to hip ratio (WHR) have been shown to be important determinants of women’s physical attractiveness, with lower BMI and WHR preferred by both men and women [3], [5]–[6], including in Malaysian populations [7]. In a recent study, Crossley et al. [8] found that the ideal own body shape preferred by men was similar to women participants’ ideal partner body, and vice versa, suggesting a consistency in the preferred ideal BMI and body shape across both genders. Both men and women preferred a relatively lower BMI with a more curvaceous body for females, while a slightly heavier muscular V-shaped body was preferred for males.

Moreover, weight appears to correlate with measures of health. For example, over-weight and obese individuals are more likely to report having asthma, headaches, back-pains and more visits to their medical practitioners [9]. They are also at a higher risk of developing hypertension, diabetes mellitus, and cardiovascular sequelae [10]–[11]. Underweight individuals, on the other hand, have reduced immunity due to malnutrition or under nutrition and are therefore more vulnerable to diseases [12]. Recent work has also found evidence that perceived facial adiposity provides a valid cue to health, reflecting susceptibility to some types of infection, and coronary health [13].

These preferences do however appear to change across different cultures, with men in areas of food scarcity or low socioeconomic status (SES) finding relatively heavier women attractive. For example, a study by Swami and Tovée [7] showed that men from rural areas preferred women with higher BMI than men from industrialized or semi industrialized areas. Additionally Tovée et al. [14] found that South African Zulu men preferred higher BMI women as being optimally attractive, while Zulu men who moved to the UK and British born African men had similar attractiveness preferences as UK Caucasian participants. Moreover, Swami et al. [15] found that sociosexually restricted and unrestricted men also differed in their preferences for the most healthy and attractive weights with unrestricted men preferring a lower weight for attractiveness. However, these studies have tended to involve rating bodies from a limited range of photographs, with obscured faces.

A recent study Coetzee et al. [16] found that females differentiate attractiveness from healthy appearance by preferring a significantly lower apparent weight in the face as being optimally attractive than healthy-looking. They discussed this difference in relation to sociocultural factors such as exposure to media, which contributes to strengthening or weakening the relationship between the most attractive and healthy looking weights. Theoretical models examining the mechanisms by which these factors influences this relationship have focused on the roles played by peers, media, and pressure from family as being responsible for individuals internalizing societal messages about the importance of thinness as a means of looking more attractive [17]–[19].

### The Role of Sociocultural Factors

Research focusing on the influence of different sociocultural factors has suggested that females are influenced to a greater extent than males, and as a result report greater levels of body dissatisfaction and lower self-esteem [20], sometimes leading to eating disorders such as bulimia or anorexia [17], [21]. McCabe & Ricciardelli [20] found that adolescent girls were less satisfied with their bodies and therefore more likely to follow strategies of losing weight while the boys were more likely to adopt strategies of gaining weight and muscle tone. Interestingly it was also seen that parent and peer feedback on weight was greater for females. Field et al. [22] found that parents and media strongly influenced the development of weight concern in individuals, leading to weight control practices like dieting, with females again showing greater weight concerns and becoming more constant dieters. Although this study found no evidence for the role of peer pressure on weight concerns, other studies suggest that peer influence is important. Stice et al. [23] assigned young women to one of two groups where a thin, attractive confederate grumbled about how fat she felt and spoke of the amount of effort she puts into losing weight, or spoke about a neutral topic. Those in the weight group reported greater levels of body dissatisfaction. Myers and Crowther [24] and Blowers et al. [25] showed that although other factors such as pressure from family and friends played an important role in mediating the thin ideal, media was a more significant reinforcer. Stice et al. [17] found that women with higher consumption of fashion, health and entertainment magazines were more likely to have internalized the thin ideal and to exhibit disordered eating.

The ideal image of men portrayed by the mass media is muscular and of normal weight, while ideal women tend to be portrayed as underweight [26]. A number of studies have investigated the effects of the thin beauty ideal (as portrayed by mass media) on the body image of women of different age groups. A meta-analytic review by Groesz et al. [27] found body image to be significantly more negative following exposure to thin media images rather than after viewing plus size models, average size models or inanimate objects. This effect was also seen to be stronger in younger females (below 19 years). Similar results were found for both men and, more strongly, women by Ogden and Mundray [28]. Cattarin et al. [29] showed that comparisons between self and media images of slim, attractive models enhance levels of dissatisfaction. Glauert et al. [30] found that a brief exposure to extremely thin or overweight bodies influences women’s perception of body normality and body ideals. They also found that exposure to thinner bodies resulted in greater body dissatisfaction and internalization of the thin western ideal. Exposure to attractive female faces also reduced self-rated attractiveness ratings of women [31]. Exposure to positive images of obese women may however reduce stigmatizing attitudes [32], and individuals who are themselves overweight are less likely to ascribe negative stereotypes to obese people [33]. Re et al. [34] found an increase in preferred facial adiposity for attractiveness in participants after viewing heavier bodies, but the decrease in preferred facial adiposity after viewing lighter bodies was found not to be significant. Boothroyd et al. [35] used full body images and found similar results, however their results also showed a significant decrease in preferred BMI following exposure to models of lower BMI.

We expect, therefore that participants who are given short-term exposure to attractive images of plus size models, or less attractive images of light weight models may prefer heavier female bodies as healthier and more attractive, while participants exposed to attractive images of light weight models, or less attractive images of plus size models, may prefer lighter female bodies as healthier and more attractive. Further, we hypothesize that preferred BMI will be positively related to participants’ own BMI.

Since media portrays a lower weight as being attractive for females and they internalize media’s message to a greater extent than males, we expect this trend to be stronger in females [36]–[38].

Here, we allow male and female participants to manipulate the apparent BMI of photographs of female bodies to make them look as healthy and, separately, as attractive as possible. We examine the influence of exposure to images of attractive and less attractive light weight and plus size models directly, by assigning participants to groups in which they are exposed to images of models of varying body weight and attractiveness. We also examine the relationship between own BMI and preferred BMI.

## Methods

All work was approved by the ethics committee of the University of Nottingham Malaysia Campus. All participants gave written informed consent prior to taking part in the study.

### Stimuli

30 female Malaysian Chinese participants (aged 18–23) were recruited from the University of Nottingham Malaysia Campus. They were asked to wear a standard, tight fitting, grey tank top and bicycle shorts so that the body shape of each individual was visible, whilst preserving appropriate modesty for the cultural setting. Height and weight were measured and used to calculate BMI [weight in kg/height in m^2^], using a Tanita SC-330 body composition analyzer. (Mean BMI:20.41, BMI range:16.4–27.7). A full body photograph of the front view of each individual was captured. Photography took place within a booth painted with Munsell N5 grey paint that was located in a room with no other lighting. The booth was illuminated with nine D50 fluorescent tubes in high frequency fixtures (Verivide, UK) to reduce the effects of flicker. The camera (Nikon d3100) settings (exposure, custom white balance, ISO) were held constant. Participants wore no makeup, had their hair pulled back and maintained a neutral expression when their photograph was taken.

**Figure 1:**
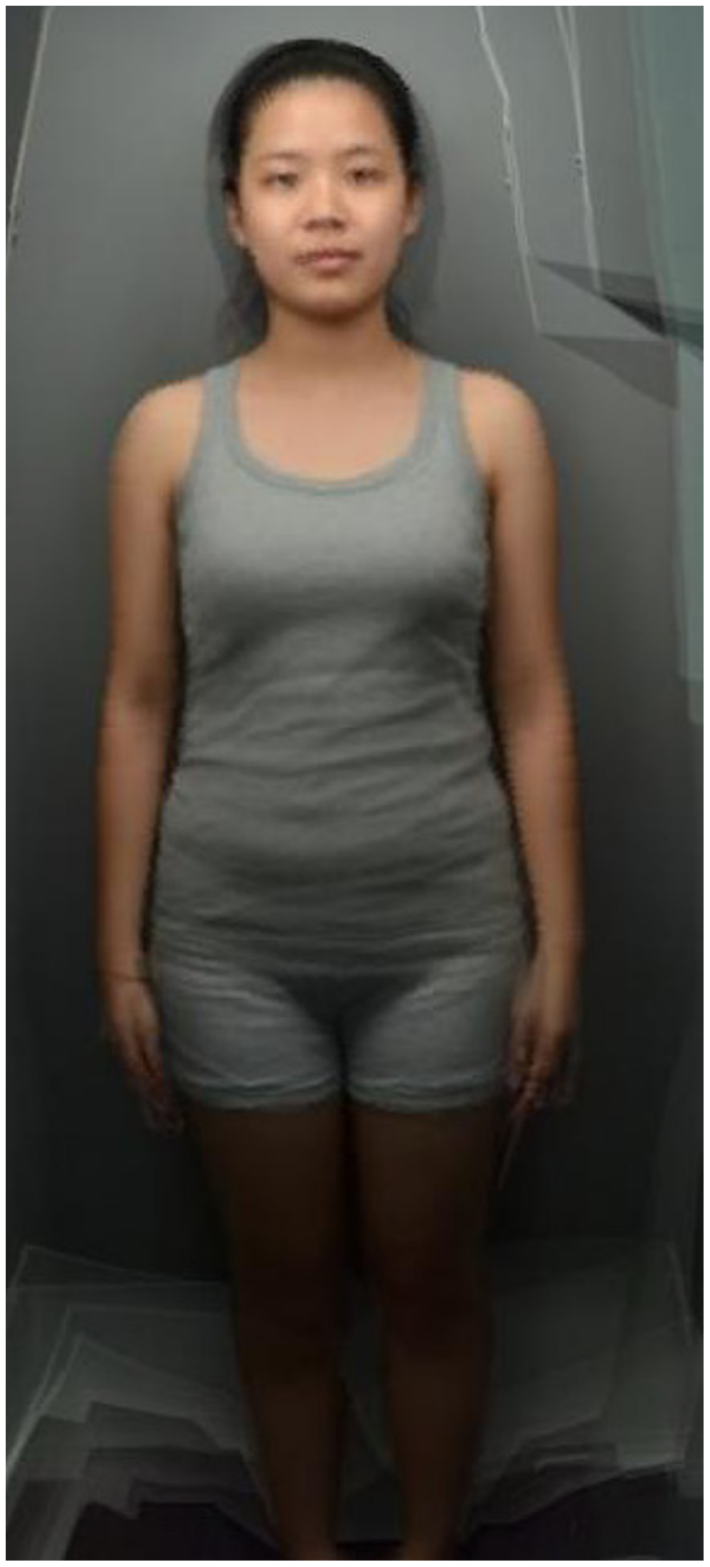
Composite image produced by averaging images of three individuals.

**Figure 2:**
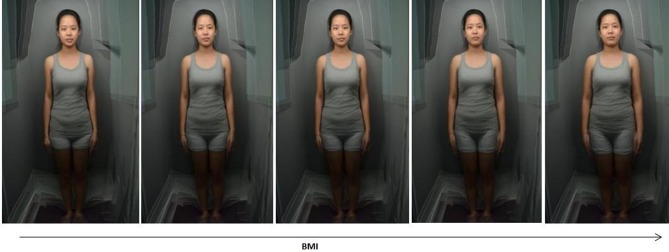
Example of transformed composite image. The left image is reduced by 5.73 units of BMI, the middle image is the original composite image, and the right image is increased by 5.73 units of BMI.

The images were then resized to 466×699 pixels, and aligned on the eyes using Adobe Photoshop CS and Psychomorph software respectively. Images were delineated by defining 252 feature points, and 10 composite images, each comprising 3 pseudorandomly selected (so that each individual only appeared in one composite image) individuals were produced, in order to mask identity (figure 1). Images of individuals with the 10 lowest and highest BMIs were then separately used to create two averaged prototype images. These two images were used to create transforms in 13 frames, in which frame 0 was reduced in apparent BMI by 5.73 units, 6 was the original image and frame 12 was increased in apparent BMI by 5.73 units (figure 2). Only shape was manipulated while skin color and texture were kept constant (following [16] and [34] but including the full body). Images were presented in an applet, in which moving the mouse horizontally cycles through the frames and changes the apparent BMI.

Model images were obtained from the websites of modeling agencies (including one specializing in plus size models), as well as Google Images. All images consisted of either full or three quarter body pictures of fashion models, and it was ensured that the model was the sole focus of the image. The face and body shape of all the models were clearly visible. The models were of varying ethnicity, and varied in their clothing (e.g.: dresses, t-shirts, pants and skirts etc.) and their pose (although none held sexually provocative poses). All models were standing except for one in the plus size less attractive group. We used images of models from websites of modeling agencies, although they may not be representative of media images in Malaysia, as incorporating models in television commercials or on billboards as stimuli could bias ratings of attractiveness and health due to familiarity with the models themselves or the products they advertise.

All images were re-sized to 366×550 pixels. Images were then rated as either “light weight” or “plus size”, and rated for attractiveness on a 10 point Likert-type scale by a separate group of 20 participants (10 men). Prior to rating, participants were presented with a slide show of all models to ensure they were aware of the range of attractiveness and weights of models in the images, to encourage raters to use the full range of the rating scale. Images were presented using Psychopy 17.0 in a randomized order. The images remained on the screen until participants responded.

Based on these judgments the images of the models were assigned into 1 of 4 groups. Images judged to be plus size and given an attractiveness rating of 6 or more fell in to the plus size- attractive group, while images judged as plus size and were given an attractiveness rating of 4 or below fell into the plus size-less attractive group. Models judged to be light weight and given an attractiveness rating of 6 or above were categorized as light weight-attractive, and those who were judged to be light weight and were given an attractiveness rating of 4 or below were categorized as light weight-less attractive. 12 images were allocated to each group.

### Procedure

We recruited 95 participants (46 men, 49 women) from the University of Nottingham Malaysia Campus. All verbally self-reported Asian ethnicity. The height and weight of participants were measured and used to calculate BMI. Participants were then assigned to 1 of the 4 model image exposure groups; plus size–attractive (8 men, 15 women, age: *range = *20–23, *M* = 20.78, BMI: *range* = 15.92–29.74, *M* = 22.54), plus size-less attractive (12 men, 12 women, age: *range = *18–24, *M* = 20.92, BMI: *range* = 16.77–31.86, *M* = 22.73), light weight-attractive (13 men, 10 women, age: *range = *19–23, *M* = 20.74, BMI: *range* = 15.71–30.38, *M* = 21.53), light weight-less attractive (13 men, 12 women, age: *range = *18–23, *M* = 20.72, BMI: *range* = 18.19–29.87, *M* = 21.87). Participants were presented with a slide show made up of the 12 models falling into the assigned category, according to the group. Images remained on screen for 5 seconds each. Participants were then presented with the BMI transforms previously created twice and instructed to manipulate the images by moving the mouse horizontally to make the image look optimally attractive and, separately, healthy. The order in which attractiveness and health judgments were made were counterbalanced across participants such that half of them made attractiveness judgments first and the other half made health judgments first. The direction of the transform was randomized, such that in some trials moving the mouse left reduced the apparent BMI, and in other trials, moving the mouse left increased the apparent BMI of the stimuli. The location of the midpoint was randomized, and the order looped so that it was not possible to easily identify the midpoint.

## Results

Analysis was conducted using SPSS version 20.0. A univariate ANOVA revealed no significant differences between groups for age (*F*_(3,91)_ = .39, *p* = .76) or BMI (*F*_(3,91)_ = .75, *p* = .53).

Linear mixed modeling was conducted to examine the difference between the BMIs chosen for attractiveness (M±SD = 17.26±2.09) and health (M±SD = 18.03±2.22) and the influence of media on these judgments (dependent variable: BMI chosen; fixed factors: media exposure group, rating type (attractiveness, health) and gender; random factor: participant ID). All main effects (group, rating and gender) were included in the model. Participant ID was nested within group and gender to avoid pseudo replication. The following interactions were also included; rating×group, gender×rating, gender×group, and gender×rating×group.

Participants chose significantly lower apparent BMI for the attractiveness than health condition (*F*_(1,1797)_ = 149.4, *p*<0.001, η*_p_* ^2^ = .077). There were significant interactions between rating×group (*F*_(3,1797)_ = 6.63, *p*<.001, η*_p_* ^2^ = .011), gender×rating (F_(1,1797)_ = 6.13; p = .013; η*_p_* ^2^ = .003) and gender×rating×group (*F*_(4,1797)_ = 9.48, *p*<.001, η*_p_* ^2^ = .016), indicating that preferred BMI was influenced by the type of model images participants were exposed to. No significant main effect of gender (*F*_(1,87)_ = 1.31 = *p* = .26, η*_p_* ^2^ = .015) or group was found (*F*_(3,87)_ = .98, *p* = .40, η*_p_* ^2^ = .033). The interaction between gender and group was also found not to be significant (*F*_(3,187)_ = .41, *p* = .75, η*_p_* ^2^ = .014).

To examine these interactions, linear mixed modeling was performed separately for men and women. (DV = BMI chosen; Fixed factors = group and rating; Random factor = participant ID). Main effects (rating and group), and interactions (rating×group) were included in the model. Participant ID was nested within group to avoid pseudo replication.

**Figure 3:**
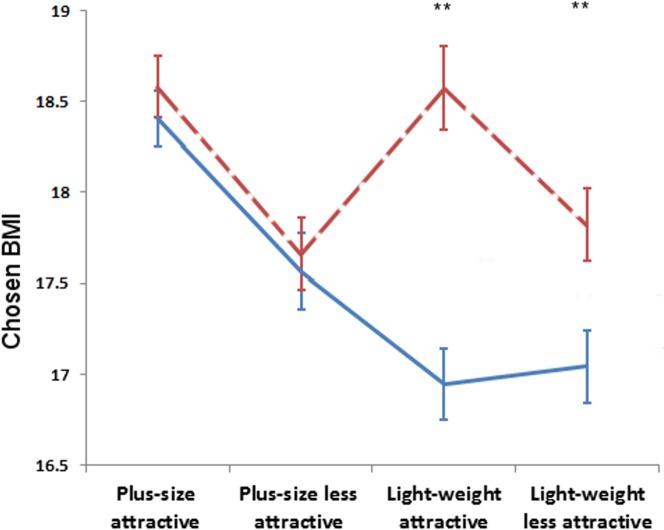
Mean chosen BMI for attractiveness (blue, solid line) and health (red, dashed line) made by women. Error bars show standard error of the mean. Asterisks indicate the significant difference between preferred BMI for attractiveness and health (* p<.05; ** p<.001).

**Figure 4:**
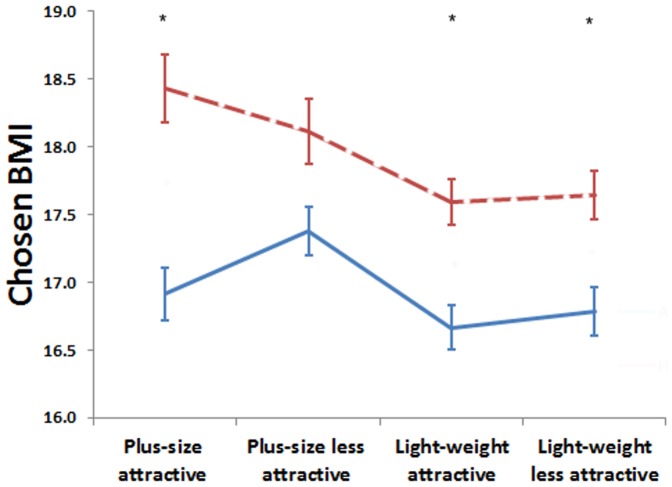
Mean chosen BMI for attractiveness (blue, solid line) and health (red, dashed line) made by men. Error bars show standard error of the mean. Asterisks indicate the significant difference between preferred BMI for attractiveness and health (* p<.05; ** p<.001).

A significant main effect of rating was found for both women (*F*_(1,927)_ = 53.92, *p*<.001, η*_p_* ^2^ = .055) and men (*F*_(1,870)_ = 95.5, *p*<.001, η*_p_* ^2^ = .099), indicating that both men and women preferred a lower weight for attractiveness than for health (figures 3, 4). Men selected an apparent BMI of 16.93±1.94 kg/m^2^ for attractiveness and a value equivalent to 17.89±1.94 kg/m^2^ for health. Women chose a BMI of 17.57±2.1794 kg/m^2^ for attractiveness and 18.17±2.2194 kg/m^2^ for health.

**Figure 5:**
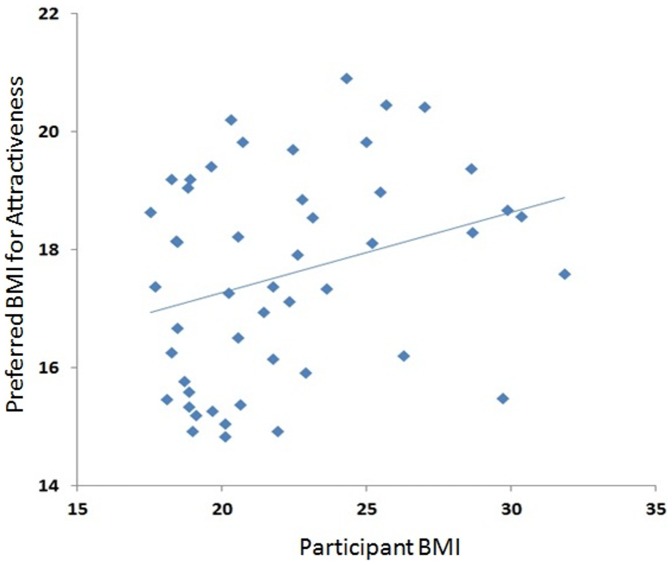
Correlation between women’s own BMI and preferred BMI for attractiveness.

The interaction between rating and group was significant for women (*F*_(3,972)_ = 13.97,*p*<.001, η*_p_* ^2^ = .043; figure 5) but not men (*F*_(3,870)_ = 2.29, *p* = .077, η*_p_* ^2^ = .008), suggesting that women’s judgments were more strongly influenced by short term exposure to model images.

Paired-sample t-tests revealed that women preferred a lower BMI in attractive than healthy trials when exposed to light weight attractive (t_9_ = 3.94; p = .003, *d* = .92) and light weight less attractive (t_11_ = 4.06; t = .002, *d* = .42) but not plus size attractive (t_14_ = .52; p = .610, *d* = .11) or plus size less attractive (t_11_ = .28; p = .783, *d* = .051) model images. Men chose lower BMI for attractiveness than health trials in light weight attractive (t_12_ = 2.86; p = .014, *d* = .68), light weight less attractive (t_12_ = 2.76; p = .017, *d* = .52) and plus size attractive (t_7_ = 3.43; p = .011, *d* = .93) conditions, and a non-significant trend in the same direction in the plus size less attractive condition (t_11_ = 1.84; p = .092, *d* = .41).

Next, we averaged the BMI chosen for attractiveness and, separately, health across all 10 transforms for each participant separately. Pearson’s correlation was then conducted between participants’ own BMI and BMI chosen for attractive and healthy appearance. Participant’s own BMI was significantly associated with their preferred BMI for attractiveness *r(49)* = 0.29, *p* = 0.041 for women, but no such effect was seen for men *r(46)* = 0.001, *p* = 0.997. For health no association was found between participant BMI and own BMI for men *r(46)* = 0–0.88, *p* = 0.56 or women *r(49)* = 0.221, *p* = 0.13.

## Discussion

In this study we aimed to see how short-term exposure to images of attractive and less attractive light weight and plus size women influenced Asian men and women’s judgments of the most attractive and healthy looking BMIs in young Malaysian Chinese women’s bodies. Results indicated that both men and women preferred a lower apparent BMI for attractiveness than for healthy appearance. Apparent BMI for attractiveness and perceived health in our sample was lower than that of previous research incorporating Caucasian face images. Preferred BMI for attractiveness and healthy appearance in our sample was also found to be within the range considered underweight by the World Health Organization (WHO). Asians have a higher percentage body fat than Caucasians of similar age, gender and BMI. As a result the WHO recently suggested that a BMI of 22–23 in Singaporeans and Hong-Kong Chinese (both urban, predominantly ethnically Chinese, Southeast Asian populations) represents a similar level of health risk as a BMI of 25 (cutoff point for being overweight) for Caucasians [39]. Aiming for a lower BMI may therefore offer health benefits for Asians.

The present study also found that men differentiated attractiveness from health by choosing a significantly lower BMI for attractiveness, while Coetzee et al. [16] did not find a significant difference between the most attractive and healthy BMI for male raters. This difference could be attributed to the difference in the type of stimuli employed. We incorporated full-length images in the current study which might have provided more information about one’s weight resulting in the discrepancy between the healthiest and most attractive BMIs for both men and women. This also suggests that judgments of female attractiveness and health are not solely based on facial cues.

Results also showed a significant interaction between rating type and group for women but not men, and that this result is driven by lower preferred BMI for attractive than healthy trials when women are exposed to light weight, but not plus size model images. This suggests that a short exposure to images of light weight models may have greater impact than plus size models, and may influence women’s judgments to a greater extent as they internalize media’s message of the ideal body more strongly than men. This supports previous studies incorporating full body images that have found weight preferences for attractiveness to be altered following exposure to images of heavy or light weight models [35]. Our findings are also in line with previous studies which have found that media images contribute strongly to lower self-esteem [40], body dissatisfaction [41]–[42] and depressive mood in women [36], [43]. It has been suggested that such factors increase the risk of developing eating disorders such as anorexia nervosa and bulimia nervosa in adolescents [44]. As a result many researchers have attempted to develop intervention programs with the aim of reducing the negative consequences created by the mass media. Wood [45] showed an improvement in eating attitudes, behaviors and body esteem scales following a media intervention program. More recently Haas et al. [46] showed that a media intervention had positive effects on female perception of their appearance.

Our results also showed that women’s own BMI influenced the BMI they perceived as being most attractive but not healthy, though it should be noted that Bonferroni correction for multiple comparisons would remove this significant result. This suggests that one’s perception of attractiveness in others may be affected by how they perceive themselves. Individuals with the lowest BMIs prefer lower weights for attractiveness, while heavier individuals prefer heavier bodies. Those within the normal BMI range may already consider themselves to be attractive and be satisfied with how they look, as their weight and body shape is more or less identical that portrayed by media [47].

It is worth noting that this study examines how a short exposure to images of models influences judgments of attractiveness and health and therefore may not generalize to the effects of more long term media exposure. That is, some participants may be exposed to more media, and different types of media, than others. This may affect judgments made, as the extent to which presentation of the models influences ratings of attractiveness and health may not be the same across all participants. Additionally, these judgments may be mediated by other psychological factors such as body dissatisfaction [26], [28], eating attitudes [48] and other sociocultural factors such as parental and peer pressure [22]–[23].

The transforms used in this study were 2D images and hence unable to capture the volumetric changes associated with changes in weight. Our sample was also restricted to female attractiveness and health while ratings were made only by college students. Therefore, future studies could incorporate male stimuli and test media’s influence on perceptions of men’s health and attractiveness. Given that media portrays a lean and muscular body, characterized by a well-developed chest and wide arms as being ideal for men [49], it is expected that the ideal BMI considered attractive for men will be similar to, or potentially even higher than, that considered healthy. In line with Crossley et al. [8], we would not expect significant differences in the ideal male BMIs preferred by men and women. These studies could also be extended to older individuals (aged 30–40 years) to see if these body ideals still remain or whether they change with time and what factors contribute to such changes. Finally, when testing these effects future studies should employ 3 dimensional full body images, as this would be a more accurate representation of one’s weight.

In conclusion, despite these limitations the results of this study show that both men and women differentiate between attractiveness and health by preferring a lower weight for attractiveness, and also that the low ‘ideal’ weight for females portrayed by media seems to influence judgments of attractiveness more than health [16] in both men and women. These results also help us to understand how exposure to images of models affects weight preferences of individuals and based on these results we can estimate the negative consequences of longer periods of exposure to media. Therefore, portraying models that are not extremely underweight (as seen in the plus size attractive group) as being attractive may help change both female and male perceptions of female attractiveness [7], [28], [47].
